# A Stretchable Electromagnetic Absorber Fabricated Using Screen Printing Technology

**DOI:** 10.3390/s17051175

**Published:** 2017-05-21

**Authors:** Heijun Jeong, Sungjoon Lim

**Affiliations:** School of Electrical and Electronics Engineering, College of Engineering, Chung-Ang University, 221 Heukseok-dong, Dongjak-gu, Seoul 156-756, Korea; jhijun000015@gmail.com

**Keywords:** electromagnetic, absorber, screen printing, stretchable

## Abstract

A stretchable electromagnetic absorber fabricated using screen printing technology is proposed in this paper. We used a polydimethylsiloxane (PDMS) substrate to fabricate the stretchable absorber since PDMS exhibits good dielectric properties, flexibility, and restoring capabilities. DuPont PE872 (DuPont, Wilmington, CT, USA), a stretchable silver conductive ink, was used for the screen printing technique. The reflection coefficient of the absorber was measured using a vector network analyzer and a waveguide. The proposed absorber was designed as a rectangular patch unit cell, wherein the top of the unit cell acted as the patch and the bottom formed the ground. The size of the patch was 8 mm × 7 mm. The prototype of the absorber consisted of two unit cells such that it fits into the WR-90 waveguide (dimensions: 22.86 mm × 10.16 mm) for experimental measurement. Before stretching the absorber, the resonant frequency was 11 GHz. When stretched along the *x*-direction, the resonant frequency shifted by 0.1 GHz, from 11 to 10.9 GHz, demonstrating 99% absorption. Furthermore, when stretched along the *y*-direction, the resonant frequency shifted by 0.6 GHz, from 11 to 10.4 GHz, demonstrating 99% absorption.

## 1. Introduction

Metamaterials are artificial structures wherein periodical unit cells are arranged infinitely such that the characteristics of the material can be controlled artificially. Owing to this extraordinary feature, metamaterials are used in cloaking technology [1], super lenses [2], antennas [3], and control components in the THz band [4].

Metamaterial absorber is a technology that uses metamaterials [5,6]. A metamaterial absorber was first introduced by Landy [7]. Owing to the infinitely periodic structure of a metamaterial absorber, the substance can be easily expanded [8]. In addition, since the absorber uses a thinner substrate than conventional absorbers (such as ferrite or wedge-tapered absorbers) [9,10], it can be fabricated in light and compact volumes.

Recently, metamaterial absorbers have been studied not only for narrow bandwidths but also for wide bandwidths through multi-layer absorbers [11], lossy patterns [12,13], frequency switching [14,15,16,17], etc. Especially, a frequency switchable metamaterial absorber can operate in a diverse spectrum. In addition, it can be used as sensors by detecting frequency changes for different physical or chemical actuation.

One of the methods of frequency switching is the use of electrical switching components such as pin diodes, varactor didoes, or microelectromechanical systems [18,19,20]. The instantaneous frequency of these absorbers is tunable owing to the use of electronic devices. However, these electrical switching components require complex direct current (DC) bias lines and are expensive. Therefore, there is a restriction on the fabrication of the absorber since unnecessary bias lines must be designed.

Alternative methods include performing frequency switching without using electrical switching components, e.g., microfluidic absorbers [15,21] or stretchable absorbers [22,23,24]. Microfluidic absorbers such as injection liquid metals [21] and crystal into microfluidic channels [15] can have tunable frequency; however, these absorbers not only are difficult to fabricate but also require complex microfluidic channel lines. Hence, we proposed a stretchable electromagnetic absorber for frequency switching. The proposed stretchable absorber can vary the resonance frequency by varying the electrical length of the resonator. Therefore, it is not necessary to design a separate electrical bias line. In addition, the stretchable absorbers can be used as remote strain sensors [25,26]. In this study, we fabricated an absorber using a screen printing technique on a stretchable polydimethylsiloxane (PDMS) material, which is expected to be applicable not only as an absorber but also as a sensor. PDMS substrate has been employed in flexible or stretchable radio frequency (RF) electronics [27,28]. Conventional fabrication of PDMS should be processed via spin-coating, via exposure to UV light, via deposition, etc. [29,30]. These are complicated processes and produce additional chemical waste. Moreover, the equipment is very expensive. In this work, we fabricated the frame of PDMS substrate using a 3D printing process, since such processes facilitate simple fabrication by eliminating the need for complicated processes. In addition, we used DuPont PE872 silver conductive ink. Since PE872 ink has not only conductive but also stretchable characteristics, it is used for the fabrication of the stretchable absorber in this study. The performance of the proposed absorber will be numerically and experimentally demonstrated. The fabrication process will be explained in the following sections.

## 2. Absorber Design

**Figure 1:**
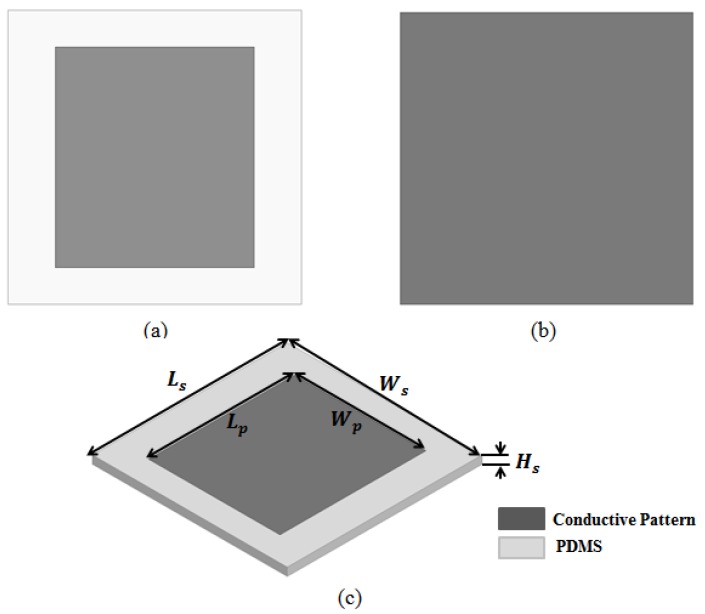
Unit cell geometry of the proposed electromagnetic absorber: (**a**) top view; (**b**) bottom view; (**c**) perspective view. *L_s_* = 12 mm; *W_s_* = 12 mm; *L_p_* = 8 mm; *W_p_* = 7 mm; *H_s_* = 0.5 mm. Conductive patterns are shown in dark gray.

The proposed electromagnetic absorber was designed as two rectangular patch unit cells. Figure 1 shows the unit cell geometry of the proposed electromagnetic absorber. The unit cell was designed to consist of a PDMS substrate of dimensions 12 mm × 12 mm × 0.5 mm and a patch of dimensions 8 mm × 7 mm, as shown in Figure 1a. The bottom of the absorber was designed to have the same width and length as the PDMS substrate, as shown in Figure 1b. The resonant frequency (*f*_0_) of the unit cell is given by the following equation [31]:

(1) $$f_{0}=\frac{c}{2\sqrt{\varepsilon_{eff}}\left\{L_{p}+0.824H_{s}\left[\frac{\left(\varepsilon_{eff}+0.3\right)\left(\frac{W_{p}}{H_{s}}+0.264\right)}{\left(\varepsilon_{eff}-0.258\right)\left(\frac{W_{p}}{H_{s}}+0.8\right)}\right]\right\}}$$

(2) $$\varepsilon_{eff}=\frac{\varepsilon_{r}+1}{2}+\frac{\varepsilon_{r}-1}{2}\left[\frac{1}{\sqrt{1+12\left(\frac{H_{s}}{W_{p}}\right)}}\right]$$

where *f*_0_ and *ε_eff_* are the resonant frequency and dielectric constant, respectively.

**Figure 2:**
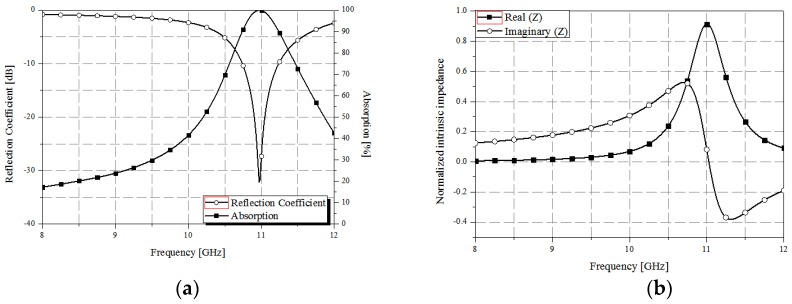
(**a**) Simulated reflection coefficient and absorption of the proposed absorber; (**b**) Normalized complex impedance of the proposed absorber.

According to Equations (1) and (2), the resonant frequency is determined by the substrate height (*H_s_*), dielectric constant ($\varepsilon_{eff}$), and the width (*W_p_*) and length (*L_p_*) of the patch. In this study, the dielectric constant and loss tangent of the PDMS substrate were obtained using the T-resonator method [32,33], and the values of these parameters were determined to be 2.89 and 0.02, respectively. The resonant frequency obtained using Equations (1) and (2) were 11 GHz. In order to validate the calculated value, we performed a resonant frequency simulation using the ANSYS high frequency structure simulator (HFSS). Simulation results confirmed a resonant frequency of 11 GHz with a reflection coefficient of −33 dB and approximately 100% absorption, as shown in Figure 2a.

The intrinsic impedance of the electromagnetic absorber was normalized to the impedance of free space. Equation (3) was used for calculating the normalized intrinsic impedance (*z*) using the *S*-parameter [34].

(3) $$z=\sqrt{\frac{{\left(1+S_{11}\right)}^{2}-{S_{21}}^{2}}{{\left(1-S_{11}\right)}^{2}-{S_{21}}^{2}}},\;\mathrm{where}\;z\;\mathrm{is}\;\mathrm{the}\;\mathrm{normalized}\;\mathrm{intrinsic}\;\mathrm{impedance}.$$

Figure 2b shows the normalized intrinsic impedance of the proposed absorber obtained from full-wave analysis. As evident from the figure, at 11 GHz, the real impedance is approximately one, and the imaginary impedance is approximately zero. In Equation (3), the transmission coefficient (*S*_21_) was assumed to be zero because the ground plane of the absorber was assumed to be a perfect conductor. Therefore, we expected to observe high absorption at 11 GHz.

**Figure 3:**
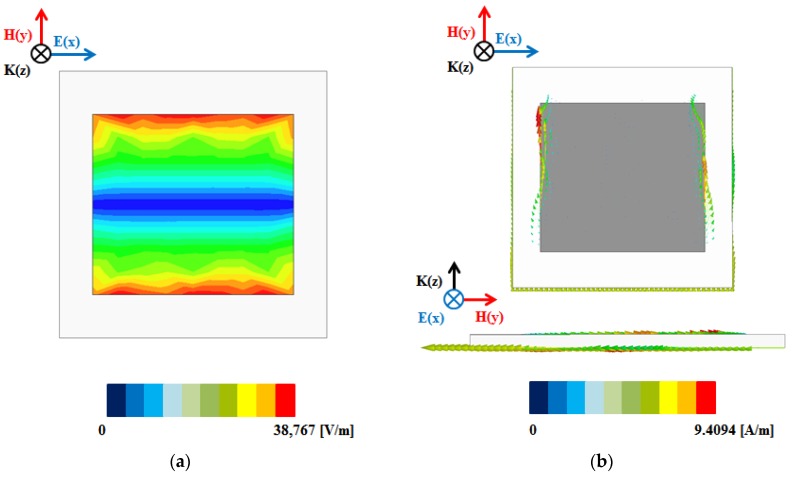
(**a**) Simulation of unit cell electric field distribution; (**b**) Vector current density.

**Figure 4:**
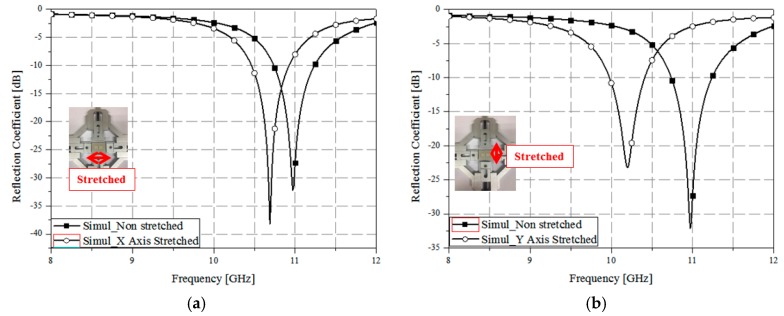
Simulated S-parameter of the absorber stretched along (**a**) the *x*-axis and (**b**) the *y*-axis.

The absorption of the electromagnetic absorber can be understood from its electrical field distribution and current density [35,36]. Figure 3a shows the magnitude of the electric field distribution of the proposed absorber at 11 GHz. It is observed from Figure 3a that the electric field is distributed at the edge of the patch which generates electric resonance. Figure 3b shows the vector current density at 11 GHz. As evident from Figure 3b, the vector current density is in the H(*y*)-direction in the top plane and in the –H(*y*)-direction in the bottom plane. In addition, the vector current densities in the top and bottom planes are anti-parallel, which generates magnetic resonance. Figure 4 shows the simulated S-parameter of the absorber stretched in different directions. When the absorber was stretched in the direction of the *x*-axis, as shown in Figure 4a, the resonance frequency shifted by 0.3 GHz, from 11 to 10.7 GHz. Furthermore, when the absorber was stretched by the same length in the direction of the *y*-axis, as shown in Figure 4b, the resonant frequency shifted by 0.75 GHz, from 11 to 10.25 GHz. Thus, we observed a wider change in frequency when we stretched the absorber in the direction of the *y*-axis.

## 3. Fabrication Processes

### 3.1. PDMS Fabrication

**Figure 5:**
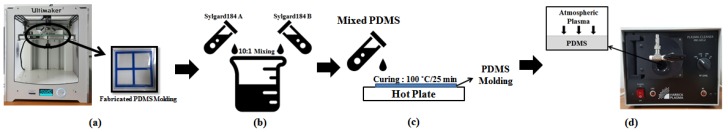
Fabrication process of the PDMS substrate; (**a**) Step 1: PDMS substrate outline, fabricated using a 3D printer; (**b**) Step 2: mixing the Sylgard184 A type and the Sylgard184 B type silicone elastomers; (**c**) Step 3: pouring PDMS into the outline and curing processing using hot plate; (**d**) Step 4: plasma treatment process.

Figure 5 shows the fabrication process of the PDMS substrate. The outline (to solidify the liquid PDMS) was manufactured on a glass substrate via a 3D printing process [37,38], as shown in Figure 5a. We used the Ultimaker2+ 3D printer (Ultimaker B.V., Geldermalsen, The Netherlands) for the outline fabrication. Subsequently, the PDMS base in liquid state and the curing agent were mixed in a beaker in a ratio of 10:1. The mixed solution was poured into the outline, as shown in Figure 5b, and cured at 100 °C for 25 min using a hot plate, as shown in Figure 5c. Subsequently, plasma treatment was performed to enhance the adhesion of the PDMS surface, as shown in Figure 5d. We used the PDC-32G plasma (Harrick Plasma, New York, NY, USA), treated at 18 W for 20 s.

### 3.2. Screen Printing Process

**Figure 6:**
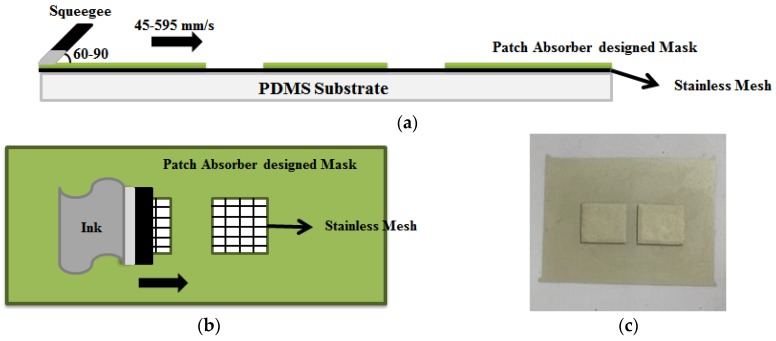
(**a**) Side view of screen printing process; (**b**) Top view of screen printing process; (**c**) Proposed fabricated prototype.

In this study, we proposed the use of screen printing technology for fabricating the top patch and bottom ground of the unit cell. The screen printing process is simple and easy, thereby allowing the fabrication of large quantities with a high number of patches and ground fabricated simultaneously. We used PE872 stretchable conductive silver ink for screen printing. Prior to screen printing, we performed plasma treatment to prevent the absorber from detaching when stretched [39]. After plasma treatment, we printed the absorber using (Daeyoung-Tech Co., Gyeonggi-do, Korea). The device provided squeegee speeds of 45–595 mm/s, and squeegee angles of 60–90°, as shown in Figure 6a,b. A stainless wire mesh with a mesh tension of 150 N and a wire count of 400 was also used in the screen printing process. We printed two unit cells on the top side of the PDMS. The gap at the top of the unit cell was 2 mm, and the size of each unit cell was 8 mm × 7 mm. The bottom ground of the cell was similarly printed. Further, a curing process was performed to improve the conductivity of the screen-printed surface. The curing process for screen printing technology can be performed via various methods such as the use of a well-ventilated oven, dryer curing, and thermal sintering [40]. In this study, we used the ON-22GW well-ventilated oven (Dongsung Science Co., Busan, Korea). The curing was performed in an oven at 100 °C for 30 min.

**Figure 7:**
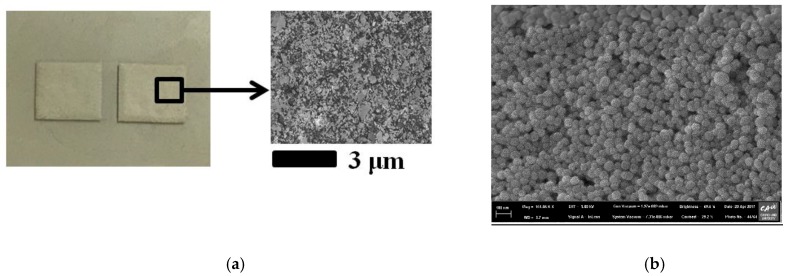
(**a**) Pictures of the screen-printed Ag pattern with drop spacing of 3 μm; (**b**) FE-SEM picture with a drop spacing of 100 nm.

We used DuPont PE872 (DuPont, USA) silver conductive ink for screen printing. When the printed thickness is 14 μm, the sheet resistivity is 0.4 Ω. Figure 7a shows the screen-printed Ag patterns, and Figure 7b shows the morphological characterization using a field emission scanning electron microscope (FE-SEM).

## 4. Measurement Results and Discussion

We used the waveguide measurement method to measure the characteristics of the fabricated absorber. The absorption *A*(ω) is given by the following Equation (4):

(4) $$A(\omega )=1-{\left|S_{11}\right|}^{2}-{\left|S_{21}\right|}^{2}=1-{\left|S_{11}\right|}^{2},\;\mathrm{where}\;A(\omega )\;\mathrm{is}\;\mathrm{the}\;\mathrm{absorption}.$$

**Figure 8:**
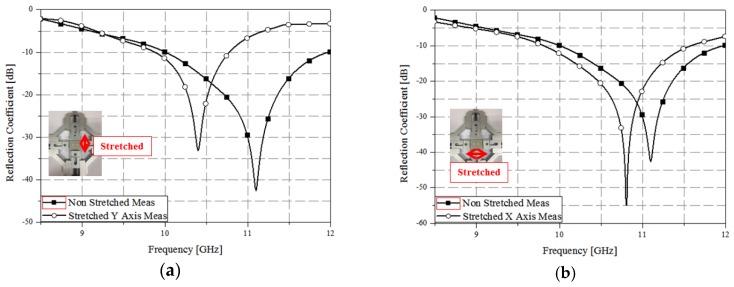
Measured reflection coefficient of the absorber stretched along (**a**) the *x*-axis and (**b**) the *y*-axis.

**Figure 9:**
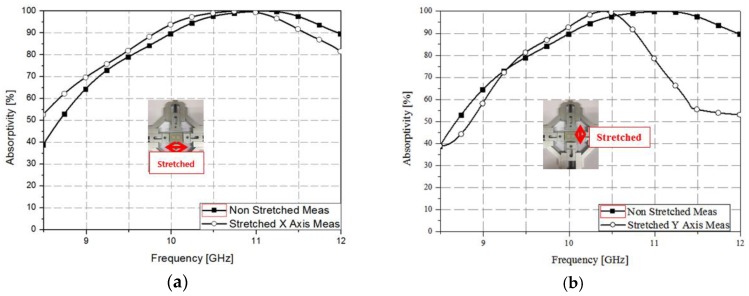
Measured absorption when stretched along (**a**) the *x*-direction and (**b**) the *y*-direction.

The transmission coefficient (*S_21_*) was assumed to be zero owing to the presence of a backside conductive plate [7]. Therefore, we measured only the reflection coefficient (*S_11_*), as shown in Figure 8, using waveguide measurements. Figure 8a shows the measurement results when the absorber was stretched in the *x*-axis direction. After stretching the absorber, it was confirmed that the resonance frequency decreased by 0.3 GHz, from 11.1 to 10.8 GHz. Similarly, Figure 8b shows the measurement results when the absorber was stretched in the *y*-axis direction. The resonant frequency decreased by 0.6 GHz, from 11 to 10.4 GHz, demonstrating 99% absorption. Therefore, it was confirmed that the frequency change was larger when the absorber was stretched in the direction of the *y*-axis, which is consistent with the simulation results. Figure 9 shows the absorption *A(ω)* calculated using Equation (4). The absorber demonstrated absorption of 99% at 10.8 GHz when stretched in the direction of the *x*-axis, as shown in Figure 9a. Furthermore, Figure 9b shows that the absorption was 99% at 10.3 GHz when the absorber was stretched in the direction of the *y*-axis.

**Figure 10:**
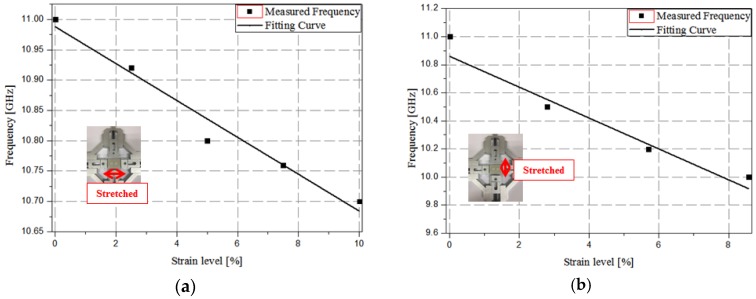
Relation between the resonant frequency and strain level along (**a**) the *x*-direction and (**b**) the *y*-direction.

Figure 10a shows the relationship between the resonant frequency and strain level along the *x*-direction. From the fitting curve of *y* = −0.0304*x* + 10.988, the sensitivity is estimated to be 3.04 × 10^7^ Hz/% when stretched along the *x*-direction. Similarly, Figure 10b shows the relationship between the resonant frequency and strain level along the *y*-direction. From the fitting curve of *y* = −0.11*x* + 10.86, the sensitivity is estimated to be 11.0 × 10^7^ Hz/% when stretched along the *y*-direction.

**Figure 11:**
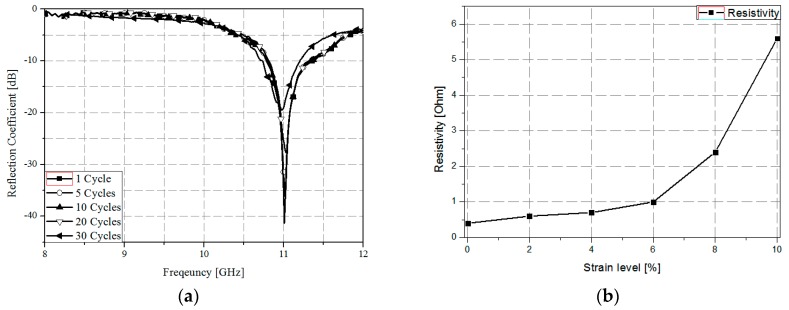
(**a**) Measured reflection coefficients after stretching for 30 cycles and (**b**) measured resistivity at different strain levels from 0 to 10%.

Figure 11a shows the measured reflection coefficients after 1, 5, 10, 20, and 30 cycles in order to prove adhesive strength and reliability. One cycle is defined as the un-stretched state after stretching the absorber. It is observed from Figure 11a that the resonant frequency is changed after 20 cycles. In addition, we measured a sheet resistance at different strain levels. As shown in Figure 11b, the resistances are 0.4, 0.6, 0.7, 1.0, 2.4, and 5.6 Ω when strain levels are 0%, 2%, 4%, 6%, 8%, and 10%, respectively.

## 5. Conclusions

In this paper, we proposed a stretchable electromagnetic absorber fabricated using screen printing technology. In order to realize stretchable characteristics in screen printing technology, a stretchable PDMS material and PE872 stretchable conductive ink were used as the substrate and conductor, respectively. Two rectangular patch unit cells of dimensions 8 mm × 7 mm were printed on top of the PDMS substrate. We measured the performance of the screen-printed electromagnetic absorber using a network analyzer and a WR-90 waveguide. The resonant frequency was determined to be 11 GHz with a reflection coefficient of −33 dB. When the length of the absorber was increased along the *y*-direction, the resonant frequency decreased by 0.6 GHz, from 11 to 10.4 GHz, demonstrating 99% absorption. When the length of the absorber was increased along the *x*-direction, the resonance frequency decreased by 0.3 GHz, from 11.1 to 10.8 GHz. Therefore, we demonstrated the successful fabrication of a stretchable electromagnetic absorber by using screen printing technology and validated the results through simulation and experiment. For practical applications, the screen-printed patterns must be encapsulated in order to protect from moisture permeation or damages.
